# Supplementary material for: Partitioning of Small Hydrophobic Molecules into Polydimethylsiloxane in Microfluidic Analytical Devices
Source: Micromachines (Basel). 2022 Apr 30;13(5):713. doi: 10.3390/mi13050713 (PMC9148048; doi:10.3390/mi13050713)
Supplement: Supplementary file 1 [file micromachines-13-00713-s001.zip › micromachines-1682818-supplementary.pdf]

Partitioning of Small Hydrophobic Molecules into Polydimethylsiloxane in Microfluidic Analytical Devices

Patrícia M. Rodrigues, Miguel Xavier, Victor Calero, Lorenzo Pastrana and Catarina Gonçalves

Supplementary Materials

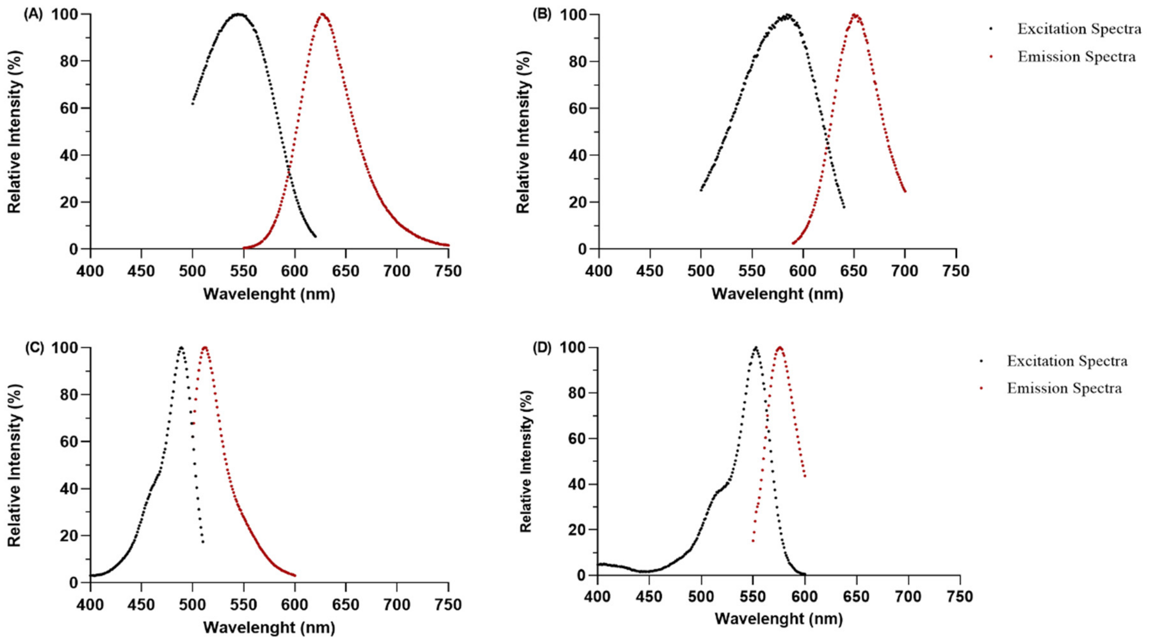

Figure S1. Graphic representation of fluorescence spectra of (A) nile red solution in ethanol; (B) nile red solution in ethanol/PBS; (D) fluorescein solution in PBS; (D) rhodamine B solution in PBS. The excitation and emission maxima of nile Red in ethanol (λex: 543 nm; λem: 627 nm), nile Red in ethanol/PBS (λex: 584 nm; λem: 650 nm); fluorescein (λex: 485 nm; λem: 515 nm) and rhodamine B (λex: 550 nm; λem: 580 nm).

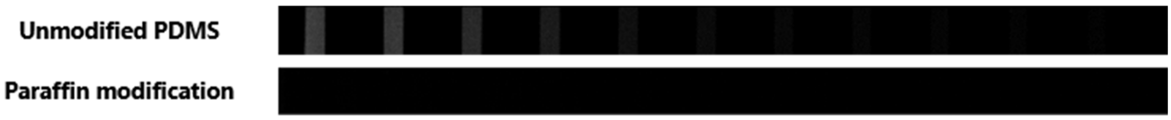

Figure S2. Confocal microscopy images of PDMS channel walls following the continuous flow of nile red in ethanol/PBS (1:3 v/v) through the microchannels for the unmodified PDMS and surface-modified with paraffin.
